# Networks of Collaboration among Scientists in a Center for Diabetes Translation Research

**DOI:** 10.1371/journal.pone.0136457

**Published:** 2015-08-24

**Authors:** Jenine K. Harris, Roger Wong, Kellie Thompson, Debra Haire-Joshu, J. Aaron Hipp

**Affiliations:** 1 Brown School, Washington University in St. Louis, One Brookings Dr., St. Louis, Missouri, United States of America; 2 Center for Public Health Systems Science, Washington University in St. Louis, One Brookings Dr., St. Louis, Missouri, United States of America; 3 Center for Diabetes Translation Research, Washington University in St. Louis, One Brookings Dr., St. Louis, Missouri, United States of America; 4 Department of Parks, Recreation, and Tourism Management, College of Natural Resources, North Carolina State University, Raleigh, North Carolina, United States of America; Northwestern University, UNITED STATES

## Abstract

**Background:**

Transdisciplinary collaboration is essential in addressing the translation gap between scientific discovery and delivery of evidence-based interventions to prevent and treat diabetes. We examined patterns of collaboration among scientists at the Washington University Center for Diabetes Translation Research.

**Methods:**

Members (n = 56) of the Washington University Center for Diabetes Translation Research were surveyed about collaboration overall and on publications, presentations, and grants; 87.5% responded (n = 49). We used traditional and network descriptive statistics and visualization to examine the networks and exponential random graph modeling to identify predictors of collaboration.

**Results:**

The 56 network members represented nine disciplines. On average, network members had been affiliated with the center for 3.86 years (s.d. = 1.41). The director was by far the most central in all networks. The overall and publication networks were the densest, while the overall and grant networks were the most centralized. The grant network was the most transdisciplinary. The presentation network was the least dense, least centralized, and least transdisciplinary. For every year of center affiliation, network members were 10% more likely to collaborate (OR: 1.10; 95% CI: 1.00–1.21) and 13% more likely to write a paper together (OR: 1.13; 95% CI: 1.02–1.25). Network members in the same discipline were over twice as likely to collaborate in the overall network (OR: 2.10; 95% CI: 1.40–3.15); however, discipline was not associated with collaboration in the other networks. Rank was not associated with collaboration in any network.

**Conclusions:**

As transdisciplinary centers become more common, it is important to identify structural features, such as a central leader and ongoing collaboration over time, associated with scholarly productivity and, ultimately, with advancing science and practice.

## Introduction

The complexity of factors associated with diabetes and ongoing translation challenges suggest that a transdisciplinary approach is needed to make progress in reducing diabetes rates projected to reach as high as one of three US adults by 2050 [1–4]. To encourage transdisciplinary approaches, the National Institute of Diabetes and Digestive and Kidney diseases (NIDDK) supports seven Centers for Diabetes Translation Research, whose mission is to “translate efficacious research findings into practice and the community to improve the health of Americans with, or at risk for, diabetes…and to enhance scientific progress and improve the uptake of research through support of rigorous translation research aimed at prevention and improved treatment of diabetes and related conditions” [5]. One of these centers is the Washington University Center for Diabetes Translation Research (WU-CDTR; http://cdtr.wustl.edu/).

As centers like the WU-CDTR seek to advance translational science, there is a growing need for evaluation of these efforts [2,3,6]. The science-of-team-science is the field concerned with understanding scientific collaboration across disciplines and its influence on scientific progress [3]. One approach used in the science-of-team-science is social network analysis (SNA) [2,7]. With a focus on relationships, SNA is well-suited to examining patterns of scholarly collaboration [8,9]. Recent advances in SNA allow researchers to go beyond traditional visual and descriptive methods to develop inferential models of collaboration in networks [9].

We identified two studies using inferential network methods to examine patterns of collaboration in large research centers as one indicator of center effectiveness [2,10]. A 2014 study by Okamoto examined 167 faculty members from multiple disciplines working across 10 centers and identified significantly higher levels of collaboration among faculty sharing the assistant professor rank (i.e., rank homophily) and an increased propensity to form ties for center leaders [10]. A 2014 study by Luke and colleagues examined growth and collaboration around scholarly products in an interdisciplinary center and found interdisciplinary network ties contributed to increases in publishing and funded grants [2]. Following these studies, we developed inferential network models to examine the influence of faculty rank, discipline, and WU-CDTR affiliation on patterns of scholarly collaboration across the WU-CDTR.

## Methods

### Data collection and management

We surveyed all members of the WU-CDTR (n = 56) about collaboration with each other and resulting scholarly products. The survey included the following items: (1) Please select the individuals you have collaborated with in a diabetes-related project in the last 12 months, and (2) What type of work have you collaborated on with the following individuals (Publication, Presentation, Grant). For the first item, the survey participant was provided with a list of all WU-CDTR members, each with a checkbox next to their name. For item 2, anyone checked in item 1 was included in a list and checkboxes were shown for each scholarly product. These two questions resulted in four distinct networks: (1) a network of general collaborations where CDTR members are linked if they had any type of collaboration, (2) a network of publication collaborations where CDTR members who worked on publishing together are linked, (3) a network of presentation collaborations where CDTR members who worked on one or more presentation(s) together are linked, and (4) a network of grant collaborations where CDTR member who worked on a grant project together are linked.

We also collected archival data on the number of years each network member had been affiliated with the WU-CDTR, academic rank, discipline of highest degree, organizational affiliation, and WU-CDTR member type (provisional, full). We sent the web-based survey on November 12, 2014 and completed data collection on December 9, 2014. Forty-nine WU-CDTR members responded (87.5%). For each pair of network members, if one or both indicated a collaborative relationship, the pair was considered linked. For example, if A indicated working with B on a publication, A and B were considered linked in the publication network regardless of whether the B confirmed the tie. Network members who did not confirm ties may have participated in the survey or not; retaining those the who did not respond to the survey is a recommended strategy resulting in closer approximation to the true network structure than dropping these network members, especially when response rates are high [11,12]. We used traditional and network descriptive statistics, network visualization, the E-I index [2], and exponential random graph modeling (ERGM) [9] to evaluate the network.

### Ethics statement

This study was approved as exempt by the Washington University in St. Louis Institutional Review Board (IRB #201410164). As part of the review, the Institutional Review Board approved the consent process. The consent process consisted of a written statement in the invitation to participate; participation in the web-based survey implied consent to use data submitted by the participant.

### Data analysis

Descriptive network statistics included density, degree centrality, and degree centralization. Network density is a ratio of existing ties to possible ties, ranging from 0 (no ties exist) to 1 (all possible ties exist). A meta-analysis of workplace networks found that those with higher density may be more productive [13]. Degree centrality is the number of ties a network member has and degree centralization is a measure of the extent to which the network is centered around one member or a small group of members who have more ties than others [14]. Degree centralization is computed $C_{deg}=\frac{\sum_{i=1}^{n}d_{max}-d_{i}}{d_{max}}$ where n is the number of nodes in the network and d or deg stands for degree. Workplace networks centralized around leadership are more productive than less centralized networks [13].

The E-I index is an indicator of the proportion of ties in the network between members of different groups compared to the total number of ties in the network. In this case, the E-I index was computed by subtracting the number of ties in the WU-CDTR network between faculty members in the same discipline from the number between two faculty members in different disciplines with the result being divided by the total number of ties. The result was between -1 and 1, with -1 indicating all ties are within discipline and 1 indicating all ties are between disciplines. Following Luke and colleagues [2] we used this metric to identify the proportion of ties in each network that are between disciplines (transdisciplinary) compared to within discipline. We computed the E-I index for each of the four networks and for each discipline within each network.

Finally, we used ERGM to estimate the probability of a collaborative tie between WU-CDTR members based on their characteristics and overall network structures. We built the models in four steps. Following Goodreau and colleagues [9,15], we started with null models. Because WU-CDTR affiliation provides opportunities to network and encourages collaboration, we added WU-CDTR affiliation type (full member or provisional member) and number of years affiliated with the WU-CDTR. Following Luke and colleagues [16] we also added an indicator of WU-CDTR leadership, coded as 1 for the director and 0 for all others. Third, we added main effects and homophily terms for faculty rank and discipline. In network models, main effects terms indicate how much more (or less) likely than expected a network member with the given characteristic is to form a tie and homophily terms indicate how much more likely two network members who share a characteristic (e.g., both in the same discipline) are to form a tie with each other compared to two network members who do not share the characteristic. So, main effects terms for faculty rank and discipline indicate whether faculty rank and faculty discipline are associated with formation of collaborative ties. For example, are senior faculty members more likely to form ties than junior faculty?

Observed networks differ from randomly generated networks with the same size and density in several ways including: (1) distribution of degree and (2) amount of transitivity [9]. Specifically, random networks have randomly distributed ties, resulting in most network members having approximately the same number of ties to others in the network. In observed networks, the distribution of links often has an exponential decline, that is, there are a small number of well-connected network members with a lot of links and many network members with few connections. Observed networks also often have more transitivity, or the friend-of-my-friend-is-my-friend property. That is, network members who are connected to each other tend to be connected to the same other network members. To account for these features, network models often include geometrically weighted terms for the distribution of degree (GWD) and transitivity (geometrically weighted edge-wise shared partnerships [GWESP]) [15]. We added these two structural terms to the model. Using the Aikake Information Criterion (AIC), we assessed fit at each stage of model-building to select the best fitting models as our final models.

Once we selected final models, we examined fit with observed data by simulating 100 networks from each of our models and comparing the simulated networks to the observed networks [9,16]. We examined how well the distribution of degree and the amount of two types of transitivity (edgewise shared partnerships and dyadwise shared partnerships) from the observed network was captured by the simulated networks. Edgewise shared partnerships occur when two people in a network who are linked are also both linked to the same third person. Dyadwise shared partnerships occur when two people in a network who are linked or unlinked are both also linked to the same third person. For example, the observed overall collaboration network included seven network members with degree = 1; if more than 95% of the simulated networks included seven network members with degree = 1, the model accurately captured this characteristic. We computed a percentage representing the proportion of correctly captured network characteristics to demonstrate model goodness-of-fit (GOF) and graphed the observed and simulated values to demonstrate fit. We used a traditional alpha (significance) level of .05 to determine which predictors in the final models were statistically significant associated with collaboration in the network. The alpha of .05 corresponds to a 95% confidence interval and odds ratios of 1 suggest no association. Therefore, odds ratios with 95% confidence intervals not including 1 were considered statistically significant and interpreted as such.

Finally, given that publishing, presenting, and grant activities are types of collaboration, and researchers working as part of the same center may collaborate on more than one type of scholarly product, we would expect some correlation among the networks. To determine whether the networks were too highly correlated to warrant separate examination, we computed correlation coefficients for each pair of networks. Using the network correlation coefficient r-QAP, we found that the overall network was strongly correlated with the publication network (r-QAP = .79) and the grant network (r-QAP = .74), but moderately correlated with the presentation network (r-QAP = .51). The publication, grant, and presentation networks were moderately correlated with each other with r-QAP ranging from .57 to .61. While these correlations indicate the networks have some similarity, there is enough difference to examine the four networks separately.

## Results and Discussion

At the time of data collection, the network included 56 members in nine disciplines (Table 1). Medicine (21.4%), psychology (8.9%), and other medical disciplines (5.4%) made up more than a third of the network, however, the majority of network members were in social sciences and education (Table 1). On average, network members had been affiliated with the WU-CDTR for 3.86 years (s.d. = 1.41). Just over half were full members and 60.7% were junior faculty.

**Table 1:** Characteristics of the WU-CDTR membership.

	m	s.d.
Years affiliated with WU-CDTR	3.86	1.41
	n	%
CDTR affiliation type		
Full	30	53.6
Provisional	26	46.4
Employer		
Missouri School of Journalism	6	10.7
National Congress of American Indians	4	7.1
Washington University in St. Louis	38	67.9
Faculty rank		
Junior	34	60.7
Senior	22	39.3
Faculty discipline		
Communication	7	12.5
Education	2	3.6
Medicine	12	21.4
Other medical	3	5.4
Other social science	3	5.4
Psychology	5	8.9
Public health	14	25.0
Social work	6	10.7
Sociology	4	7.1

**Fig 1:**
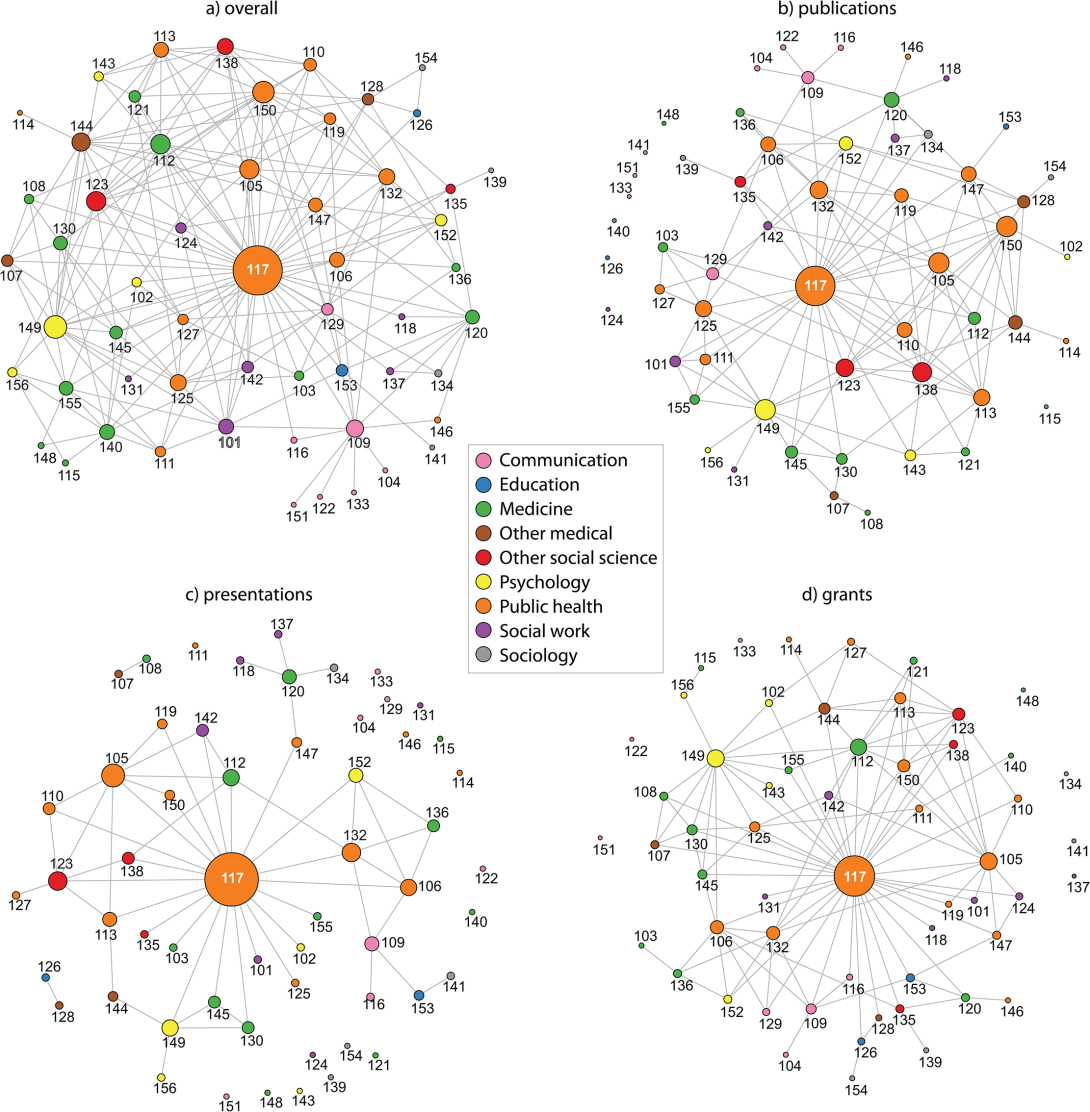
Collaborative ties among all full and provisional members of the WU-CDTR. Node size represents the number of collaborators each network member has in the network (larger nodes indicate more collaborators) and node color represents discipline.

The overall collaboration network included 209 ties among the 56 network members for a network density of .13, or 13% of possible ties (Table 2). On average, WU-CDTR members collaborated with 7.2 other WU-CDTR network members on diabetes-related projects in the past year, consistent with other work examining collaboration on translational research [2], every WU-CDTR member had at least one collaborator in the network. The most connected person in the network, the WU-CDTR director, had collaborative ties with 41 WU-CDTR members (Fig 1A).

**Table 2:** Density, centralization, and transdisciplinarity (E-I index) of the four WU-CDTR collaboration networks.

	Measure		
Network	Density	Centralization	E-I index
Overall	**.13**	**.61**	.48
Publication	.09	.39	.47
Presentation	.04	.36	.39
Grant	.08	.58	**.49**

The network formed by collaborating on publications included 131 connections for a density of .09, or 9% of possible ties (Fig 1B). On average, network members collaborated on publications with 4.7 others in this network. Eight people in the network were isolates with no connections; the highest number of connections was 26. The presentation network had 59 connections for a density of .04, or 4% of possible ties (Fig 1C). Seventeen network members had not collaborated on a presentation with any others WU-CDTR members. The grant network had 117 connections among the 56 network members for a density of .08 or 8% of possible ties (Fig 1D). Seven network members had no connections in the grant network.

Network centralization was highest for the overall network (c = .61) and the grant network (c = .59) and lowest for the presentation network (c = .36). The E-I index for discipline ranged from .39 to .49 for the four networks indicating more external than internal ties by discipline in every network. The grant network was the most transdisciplinary with an E-I index of .49, while the presentation network was the least transdisciplinary (E-I = .39). Within discipline, network members with their highest degree in education or sociology had the highest proportion of transdisciplinary collaboration. Communication (E-I range: .20-.33) and public health (E-I range: 0-.26) were the disciplines with the lowest proportion of transdisciplinary ties.

**Fig 2:**
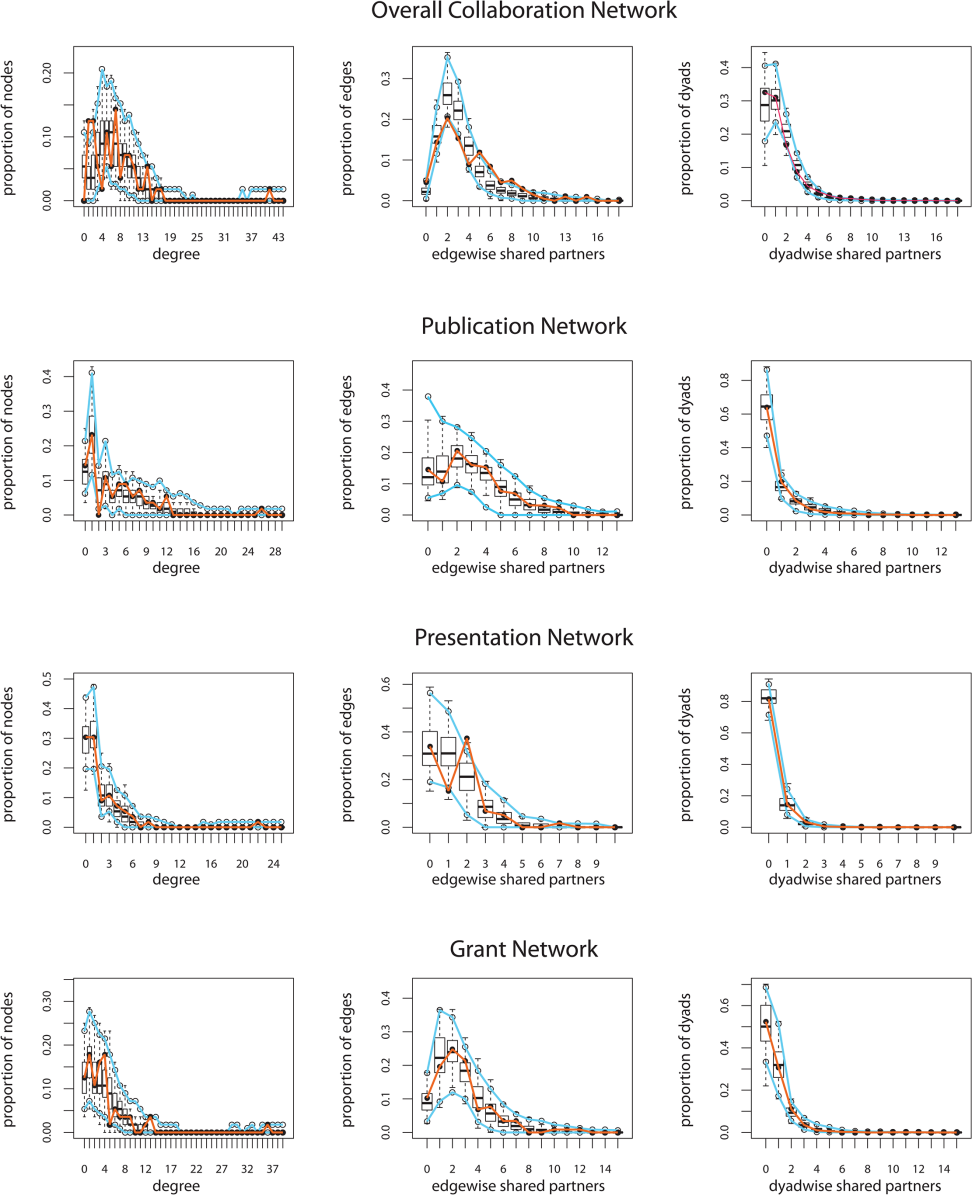
Goodness-of-fit (GOF) plots for degree, edgewise shared partnerships, and dyadwise shared partnerships for the final models for each network. For each graph, when the orange line representing the observed value of the network statistic is between the two blue lines representing the simulated networks, the networks simulated from the model captured the observed network statistic.

The full models including WU-CDTR affiliation characteristics, faculty rank, faculty discipline, and structural terms were the best fitting models. Table 3 shows the final models including the GOF percent. Fig 2 shows GOF plots with the observed networks represented by orange lines and the range of the middle 95 of the 100 simulated networks represented by the blue lines. When the orange line falls between the blue lines, the characteristic is captured by the simulated networks; when the orange line is outside the blue lines, the characteristic was not captured well by the simulated networks.

**Table 3:** Final ERGM explaining overall collaboration and collaboration on presentations, publications, and grants among members of the WU-CDTR in 2014. Estimates shown in bold are statistically significant at the .05 significance level (α = .05).

	Overall Collaboration	Collaboration on Publications	Collaboration on Presentations	Collaboration on Grants
	OR (95% CI)	OR (95% CI)	OR (95% CI)	OR (95% CI)
Edges	**.00 (.00-.01)**	**.00 (.00-.01)**	**.00 (.00-.01)**	**.00 (.00-.01)**
***CDTR affiliation characteristics***				
Years in CDTR	**1.10 (1.00–1.21)**	**1.13 (1.02–1.25)**	1.14 (.91–1.43)	1.05 (.93–1.18)
Member type (full)	1.11 (.79–1.54)	.99 (.69–1.41)	.89 (.47–1.71)	1.24 (.79–1.95)
CDTR leader^1^	**3.55 (1.74–7.24)**	**3.26 (1.58–6.71)**	**9.91 (3.90–25.17)**	**11.66 (5.15–26.42)**
***Rank and discipline***				
*Faculty rank*				
Senior faculty	1.02 (.73–1.45)	1.04 (.73–1.49)	2.04 (.92–4.52)	1.17 (.75–1.82)
Faculty rank homophily	.98 9.70–1.39)	1.09 (.71–1.67)	.75 (.38–1.48)	1.01 (.65–1.59)
*Faculty discipline*				
Medicine	ref	ref	ref	ref
Communication	.69 (.46–1.04)	.79 (.51–1.24)	.55 (.22–1.37)	.61 (.33–1.12)
Education	.95 (.52–1.73)	.27 (.03–2.67)	1.51 (.49–4.65)	1.15 (.55–2.40)
Other med	1.43 (.97–2.11)	1.36 (.89–2.07)	.94 (.37–2.40)	1.14 (.71–1.84)
Other social	1.38 (.95–2.00)	1.44 (.97–2.15)	1.69 (.85–3.38)	1.34 (.82–2.20)
Psychology	1.11 (.79–1.55)	1.13 (.79–1.62)	1.25 (.66–2.37)	1.18 (.78–1.78)
Public health	1.06 (.83–1.35)	1.25 (.91–1.72)	1.19 (.71–2.00)	1.21 (.87–1.70)
Social work	.78 (.82–1.17)	.77 (.47–1.27)	.73 (.32–1.69)	.61 (.32–1.15)
Sociology	.53 (.27–1.04)	.68 (.31–1.46)	.50 (.13–1.93)	.24 (.06–1.13)
Discipline homophily	**2.10 (1.40–3.15)**	1.36 (.77–2.42)	1.82 (.83–3.97)	1.39 (.77–2.50)
***Structural terms***				
GWD	1.85 (.54–6.27)	**3.25 (1.23–8.60)**	2.22 (.85–5.80)	**6.38 (1.59–25.57)**
GWESP	**4.41 (2.61–7.47)**	**2.26 (2.42–3.26)**	**2.23 (1.57–3.16)**	**3.08 (2.15–4.42)**
***GOF percent***	***94*.*6%***	***99*.*3%***	***99*.*3%***	***99*.*3%***

^1^During analysis of the four networks, we found that the director of the WU-CDTR was an outlier in all networks. Specifically, the director had more links in each network compared to other network members. To determine whether we could learn more about collaboration from modeling the networks without the director, we conducted a sensitivity analysis. We removed the director from the data and re-estimated the ERGMs. For the ERGMs we compared the significance, direction, and magnitude of the associations in the models with and without the WU-CDTR director. With a few minor exceptions, we found associations to be nearly the same with or without the director. The GOF was better for two models with the director (overall and publication networks) and two without (presentation and grant networks). Given the central role of the director in the network and the lack of large differences between models accounting for the director and models removing the director, we left the director in the network for all results presented below.

Years spent in the WU-CDTR was significantly associated with overall collaboration and collaboration on publications. Specifically, for every year a network member had been involved with the CDTR, they were 10% more likely to have collaborated with another WU-CDTR member and 13% more likely to have worked on a publication with another WU-CDTR member. Length of WU-CDTR affiliation was not significantly associated with presentation or grant collaboration. Discipline homophily, or being in the same discipline, was significantly associated with overall collaboration. Specifically, two WU-CDTR members in the same discipline were more than twice as likely to form a collaborative tie as two WU-CDTR members in different disciplines (OR = 2.10; 95% CI: 1.28–2.90).

Faculty rank and discipline were not associated with tie formation in any of the networks. This indicates that junior and senior faculty and faculty from all disciplines were equally likely to participate in collaborating on diabetes-related publications, presentations, and grants once WU-CDTR affiliation characteristics were accounted for. Faculty rank homophily was also not significant in any of the networks indicating that collaborations are no more likely to occur between two faculty members of the same rank as between two faculty members of different ranks. Discipline homophily was not associated with collaboration in the publication, presentation, or grant networks; faculty members were no more or less likely to collaborate with colleagues in their own discipline compared to other disciplines on these specific scholarly products.

## Conclusions

We examined collaboration in the WU-CDTR network of scholars, which included 56 members representing nine disciplines at the time of the study. Similar to Okamoto (2014), who examined 10 centers for population health and disparities [10], we found the WU-CDTR network to be highly centralized around the director, who was significantly more likely to form ties across all types of scholarly collaboration. Given the similarity of findings in the current study and the Okamoto study, this may emerge as a defining feature of transdisciplinary scholarly networks. Researchers conducting studies in this area in the future may wish to test this network feature by examining transdisciplinary centers with multiple leaders to determine whether all leaders, or leaders with certain qualities or roles, are more likely to play central roles. High centralization may be a strength of these networks; a meta-analysis of research on team structures in workplaces found strong evidence that teams centralized around a leader were more productive [13]. As research in this area develops, it will be important to identify individual and network characteristics like centrality and centralization that are consistent across centers and may facilitate or hinder scientific progress.

Collaboration overall was significantly higher between two network members from the same discipline than those from different disciplines. However, when collaboration was specific to a scholarly product type (publication, presentation, or grant), discipline homophily was no longer a significant predictor of collaboration. This may be due to other types of collaboration among faculty members not captured by these three scholarly products, for example, faculty members may collaborate on teaching or mentoring students and faculty working in this area rather than on a publication, presentation, or grant. It is also possible that there is some inaccuracy in network member reports of collaboration; mistaken perceptions of ones’ own network [17,18] and social desirability bias might both lead a network member to report more collaborations than they actually have.

Of the three scholarly product networks, the publication network was the densest. Network density was moderately and positively associated with team productivity in a meta-analysis of social network studies of teams [13], suggesting that the publication network may be the most productive of the scholarly product networks formed by WU-CDTR members. The density of this network may stem from opportunities provided by the WU-CDTR to collaborate with other network members for the purpose of manuscript development. Specifically, in February 2014 the WU-CDTR organized a one-day event called *Next Steps*, which brought together faculty, staff, and students affiliated with the WU-CDTR to outline manuscripts addressing four priority areas for the NIDDK. Participants self-selected into working groups for each of the priority areas and spent the day working together to refine paper topics and develop paper outlines and writing groups. The WU-CDTR provided administrative and other support to working groups post-meeting as necessary to assist in manuscript development and submission. The event resulted in 13 manuscripts accepted for publication. Other research centers aiming to increase transdisciplinary collaboration might consider adopting the *Next Steps* model, providing network members with dedicated time to work together on a specific goal or product. Examining the development of collaborative ties over time might also provide additional insight into the formation of the dense publication network. Specifically, as the WU-CDTR continues to develop as a center, it may be useful to measure collaboration among its members on a more regular basis in order to identify specific changes in network structure following events like *Next Steps*.

Overall, the presentation network appeared the weakest; it was the least dense, least centralized, and had the lowest proportion of transdisciplinary ties of the networks. Given that presentations increase the visibility of a project and its results, it may be worth additional effort by WU-CDTR leadership to encourage collaboration on presentations.

Finally, length of time affiliated with the WU-CDTR was associated with an increased likelihood of collaboration overall on publications but not on presentations or grants. This may be due to an increased number of formal and informal opportunities to collaborate over time, especially on publications supported through the *Next Steps* event. As a result of this finding, the WU-CDTR is currently implementing a *Next Steps* model for grant development.

There are limitations to our work including the possible misperception of collaborative ties mentioned previously. In addition, the network was measured at a single point in time, so it was not possible to examine changes in transdisciplinary collaborations over time. As the WU-CDTR continues its work, we plan to measure the network periodically in order to better understand its development and corresponding productivity. The presentation network and grant network were relatively sparse, which resulted in a few wide confidence intervals indicating a lack of precision in these estimates. Finally, while it is important to measure proximal outcomes (e.g., papers and grants) as one indicator of center activity and effectiveness, future research on the role of large transdisciplinary research centers should also examine more distal outcomes including the role of the center in facilitating adoption and implementation of evidence-based programming. Despite these limitations, we believe this study to be the first examination of transdisciplinary collaboration in an NIDDK funded CDTR and one of the first evaluations of a large transdisciplinary academic center.

With recent increases in funding for large transdisciplinary centers like the CDTRs and the Clinical and Translational Science Awards [2] and the ongoing advances in the science-of-team-science, inferential research on the structures that form large transdisciplinary efforts is in its infancy. As work in this area develops, it is important to identify strategies like *Next Steps* and common features like network centralization associated with scholarly productivity and, ultimately, with advancing science and practice.

## Supporting Information

S1 Dataset Network data for the four networks and attribute data for the network members. (XLSX)
